# Chimeric influenza haemagglutinins: Generation and use in pseudotype neutralization assays

**DOI:** 10.1016/j.mex.2016.12.001

**Published:** 2016-12-15

**Authors:** Francesca Ferrara, Nigel Temperton

**Affiliations:** Viral Pseudotype Unit, School of Pharmacy, Anson Building, University of Kent, Central Avenue, Chatham Maritime, Kent, UK

**Keywords:** Chimeric influenza HA pseudotype production, Influenza pseudotypes, Chimeric haemagglutinin, Virus neutralization

## Abstract

Recently chimeric influenza haemagglutinins (cHAs) have been generated as potential ‘universal’ vaccination antigens and as tools to identify HA stalk-directed antibodies via their use as antigens in ELISA, and virus or pseudotype-based neutralization assays. The original methods [1], [2] used for their generation require the amplification of regions of interest (head and stalk) using primers containing *SapI* sites and subsequent cloning into pDZ plasmid. This requires precise primer design, checking for the absence of *SapI* sites in the sequence of interest, and multi‐segment ligation. As an alternative strategy we have developed and optimized a new protocol for assembling the cHA by exploiting Gibson Assembly.

• This method also requires precise primer design, but it is rapid and methodologically simple to perform. We have evaluated that using this method it is possible to construct a cHA encoding DNA in less than a week.
• Additional weeks are however necessary to optimize the production of pseudotyped lentiviral particles and to perform neutralization assays using them as surrogate antigens.
• In comparison to the original protocols, we have also observed that performing parallel neutralization assays using pseudotypes harbouring the two parental HAs, permits effective delineation between stalk and head antibody responses in the samples tested.

## Graphical abstract

**Figure:**
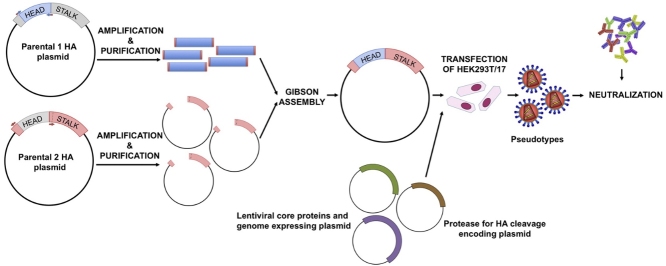

## Materials and instruments

• Haemagglutinin-expressing plasmids (*parental 1* and *parental 2*)
• Haemagglutinin sequences (nucleotide and amino acid)
• Q5^®^ High-Fidelity DNA Polymerase (New England Biolabs, cat. # M0491S)
• DreamTaq Green PCR Master Mix (Thermo Fisher Scientific, cat. # K1081 or K1082) (OPTIONAL)
• Gibson Assembly Cloning kit (New England Biolabs, cat. # E5510).
• FastDigest *DpnI* (Thermo Fisher Scientific, cat. # FD1703)
• FastDigest or conventional Restriction Enzymes (Thermo Fisher Scientific)
• RNAse/DNase free water
• Agarose
• Nucleic Acid Gel Stain
• Tris base, acetic acid and EDTA (TAE) Buffer
• GeneRuler™ 1 kb DNA Ladder Mix (Thermo Fisher Scientific, cat. # SM0314) or similar DNA Ladder
• Loading dye (Thermo Fisher Scientific, cat. # R0611) or similar loading dye
• QIAquick PCR Purification Kit (QIAGEN, cat. # 28104) or similar kits (OPTIONAL)
• QIAprep Spin Miniprep Kit (QIAGEN, cat. # 27104) or similar kits
• Luria Bertani (LB) agar plates with antibiotics appropriate to the plasmid used
• LB broth with antibiotics appropriate to the plasmid used
• Thermocycler
• Water bath or heating block
• Incubator at 37 °C
• Gel electrophoresis system
• Microwave (to dissolve agarose gel)
• Bioinformatics software for DNA and protein sequence and structure analysis

## Cloning the chimeric haemagglutinin

### Selection of haemagglutinin parental strains

Before proceeding to clone the chimeric HA (cHA), it is necessary to identify which HA subtypes/strains will be used to generate the cHA. There are different factors to take into consideration. Firstly, it is important to identify the final purpose of the project for which cHA are required. For example if human stalk-directed antibody responses are to be detected it is more appropriate to choose the head region of an HA subtype that is less related to circulating human influenza strains (i.e. H1 and H3) or other to strains that have been shown to infect humans (e.g. H5 and H7), such as H11 or H16. Furthermore, depending on the experimental requirements, selecting for the stalk an HA that is currently, or has previously circulated in humans may also be appropriate. This is extremely important since it permits the minimizing of detection of cross-reactive antibodies against the head, and the maximising of the identification of stalk-directed responses.

### Cloning strategy

**Fig. 1:**
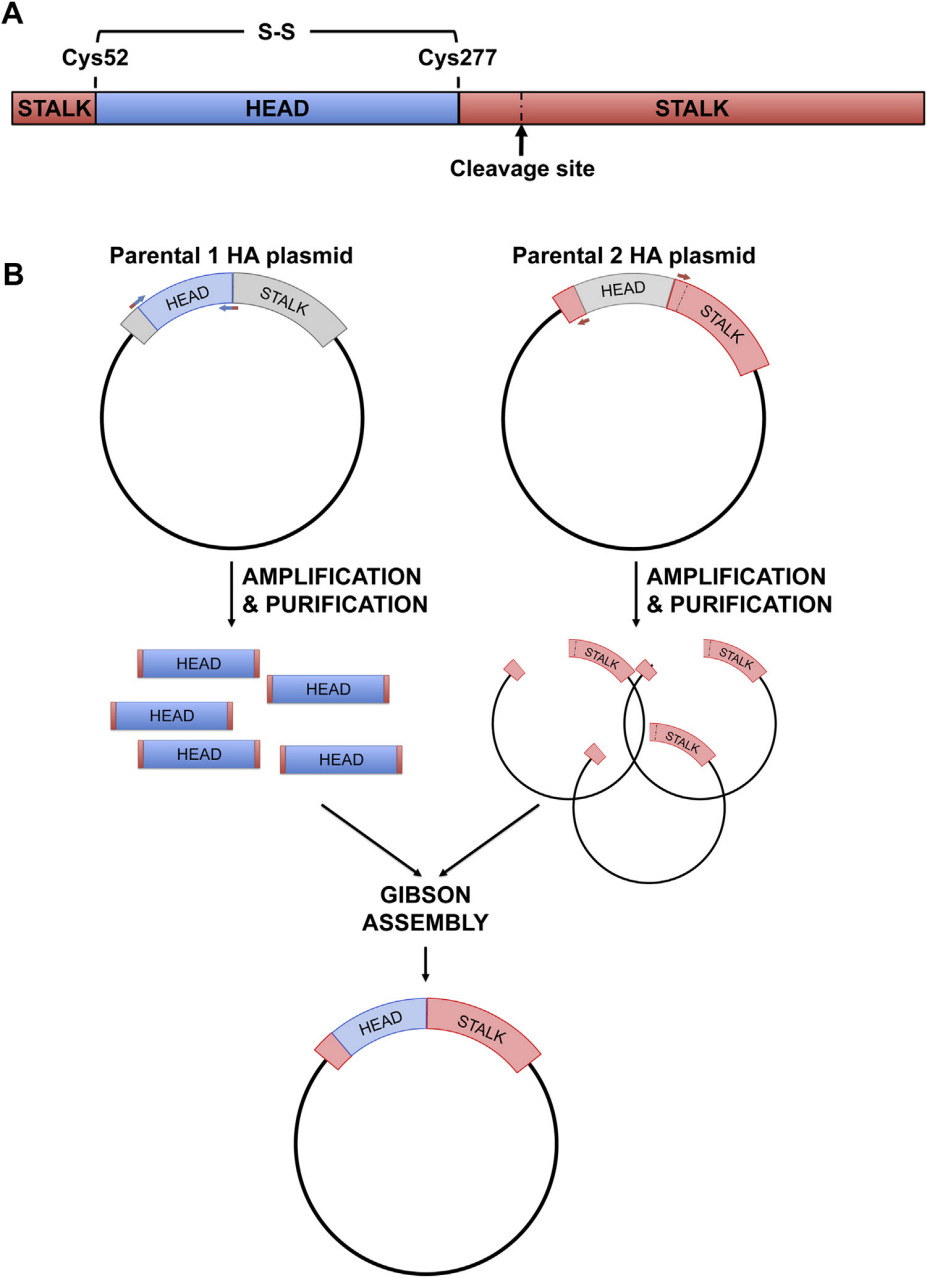
Chimeric haemagglutinin and cloning strategy to generate it. A. Structure of influenza haemagglutinin polypeptide highlighting the cleavage site, the head, the stalk, Cys52 and Cys277 that need to be identified in the HA sequences to proceed with cHA cloning; B. Cloning strategy used to build cHA using Gibson assembly method: after primer design the HA head and the HA stalk with the plasmid backbone are amplified by PCR from two different plasmids encoding the parental HAs; following purification of fragments, a Gibson assembly reaction is set up to obtain a cHA.

Two cysteines, Cys52 and Cys277 represent the hinge between the HA head and stalk regions. These two cysteines can thus be exploited using DNA recombinant technology to exchange the HA head of one virus with the HA head of another influenza strain creating cHA [2] (Fig. 1A). The protocol described here (Fig. 1B) is based on the amplification of plasmid DNA to create two linearized DNA fragments:

• The first fragment (around 700 bp depending on the influenza strain used) will correspond to the HA head region, it will be amplified from one of the HA-encoding plasmids (*parental 1*) and it will contain 5′ and 3′ additional region overlapping with the stalk of the other HA;
• The second fragment comprises the expression vector and the stalk region (length depending on the influenza strain used, around 1000 bp, and on the expression vector used), and will be amplified from the other HA-encoding plasmid (*parental 2*).

Gibson Assembly is then used to combine these two fragments. The Gibson Assembly methodology permits the rapid assembly of multiple overlapping DNA fragments through a reaction in which three different enzymes are used at the same time: an exonuclease to create 3′ overhangs, a polymerase to fill gaps, and DNA ligase to close the nicks in the assembled DNA [3].

### Step 1: Primer design

New England Biolabs, who distribute the Gibson Assembly kit used in this method, offers on its website a free tool, NEBuilder Assembly Tool, that permits in-silico assembly of the DNA sequence of interest (e.g. the cHA). This web tool additionally designs the primers that should be employed to generate the fragment of interest (HA head and vector plus HA stalk) that will be assembled.

Below we report step-by-step the procedure used to design the HA primer using this web tool.

i Align using Jalview [4] or equivalent software, the amino acid sequences of the two HAs.
ii Identify Cys52 and Cys277 in the two sequences. This step should be performed carefully since the numbering reported corresponds to the H3 numbering system and may not correspond to the numbering of the HAs used
iii After identification of the Cys, if possible, check on the structure of the HA subtypes chosen such that the Cys identified correspond to the hinge Cys52 and Cys277. These structures, if available can be downloaded from the RCSB Protein Data Bank database (www.rcsb.org) [5]. We use Swiss PDB-Viewer [6] to visualize them but it is possible to use other protein structure visualization tools (e.g. Jalview, UCSF Chimera, RasMol, Jmol, etc.).
iv Assemble the predicted amino acid sequence of the cHA by removing the amino acid sequence corresponding to the original HA head (between the two cysteines) and inserting in the amino acid sequence of the other HA. This chimeric sequence will be used to check the final assembly.
v Now that the cysteines have been identified it is necessary to identify the nucleotides that encode for them. Software such as DNADynamo (BlueTractorSoftware), or BioEdit can be useful for this purpose.
  a Once the nucleotides that encode the cysteines of the HA used for the stalk (parental 2) have been identified note down the number of the last nucleotide encoding Cys52 and the number of the first nucleotide encoding Cys277.
vi Once the nucleotides that encode the cysteines of the HA used for the head (parental 1) have been identified, copy the sequence between the last nucleotide encoding Cys52 and the number of the first nucleotide encoding Cys277 in a new sequence text file that you will conserve.

**Fig. 2:**
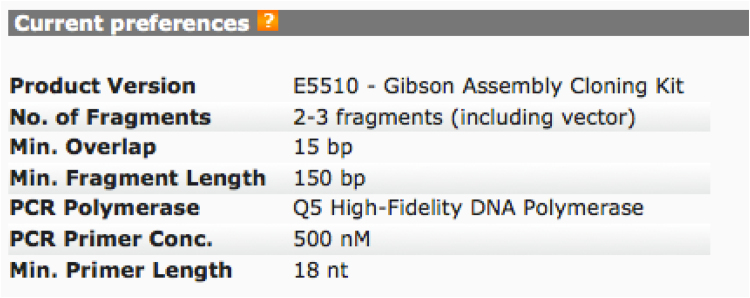
NEBuilder Assembly Tool preferences.

**Fig. 3:**
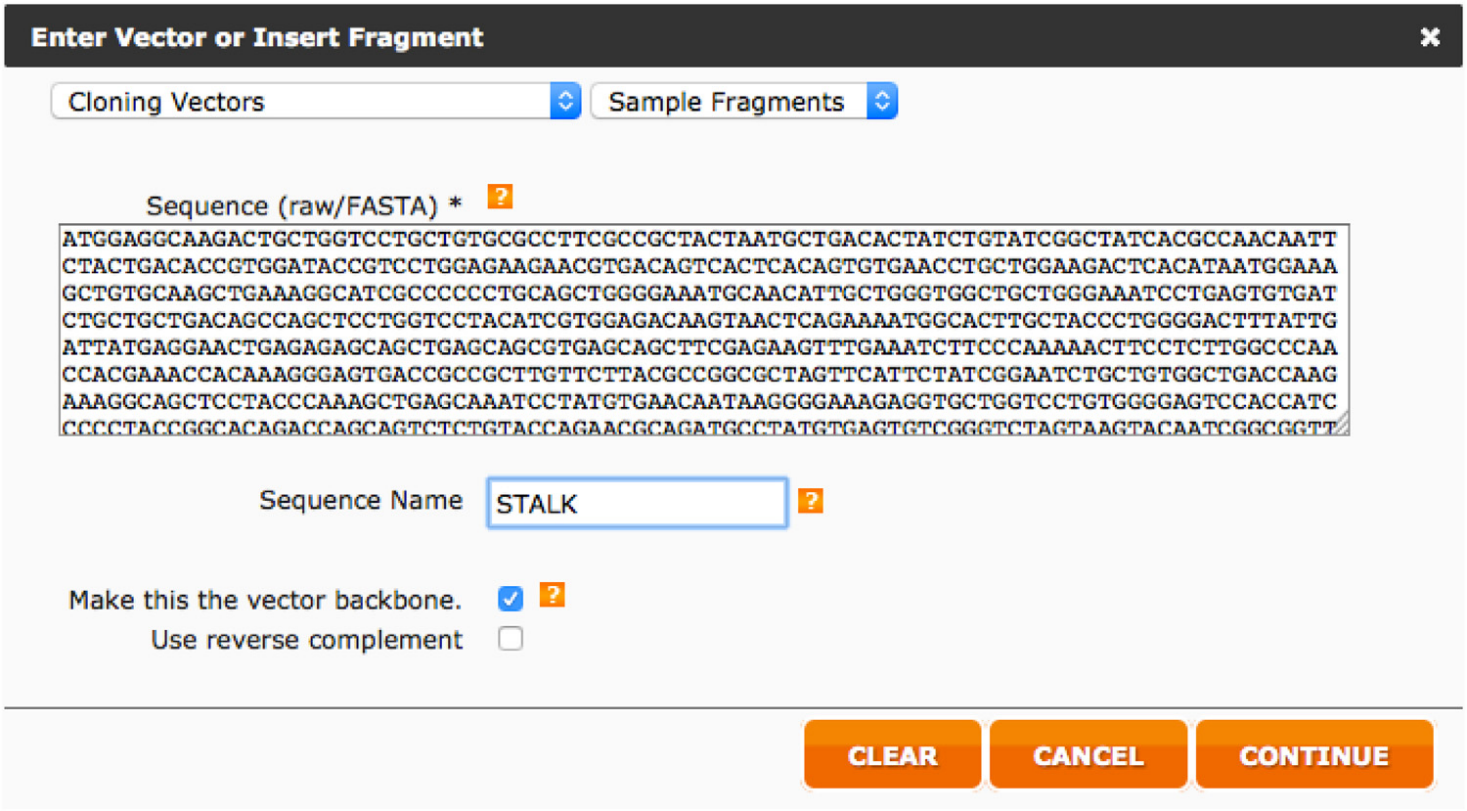
Entry of the parental 2 HA (stalk donor) sequence.

**Fig. 4:**
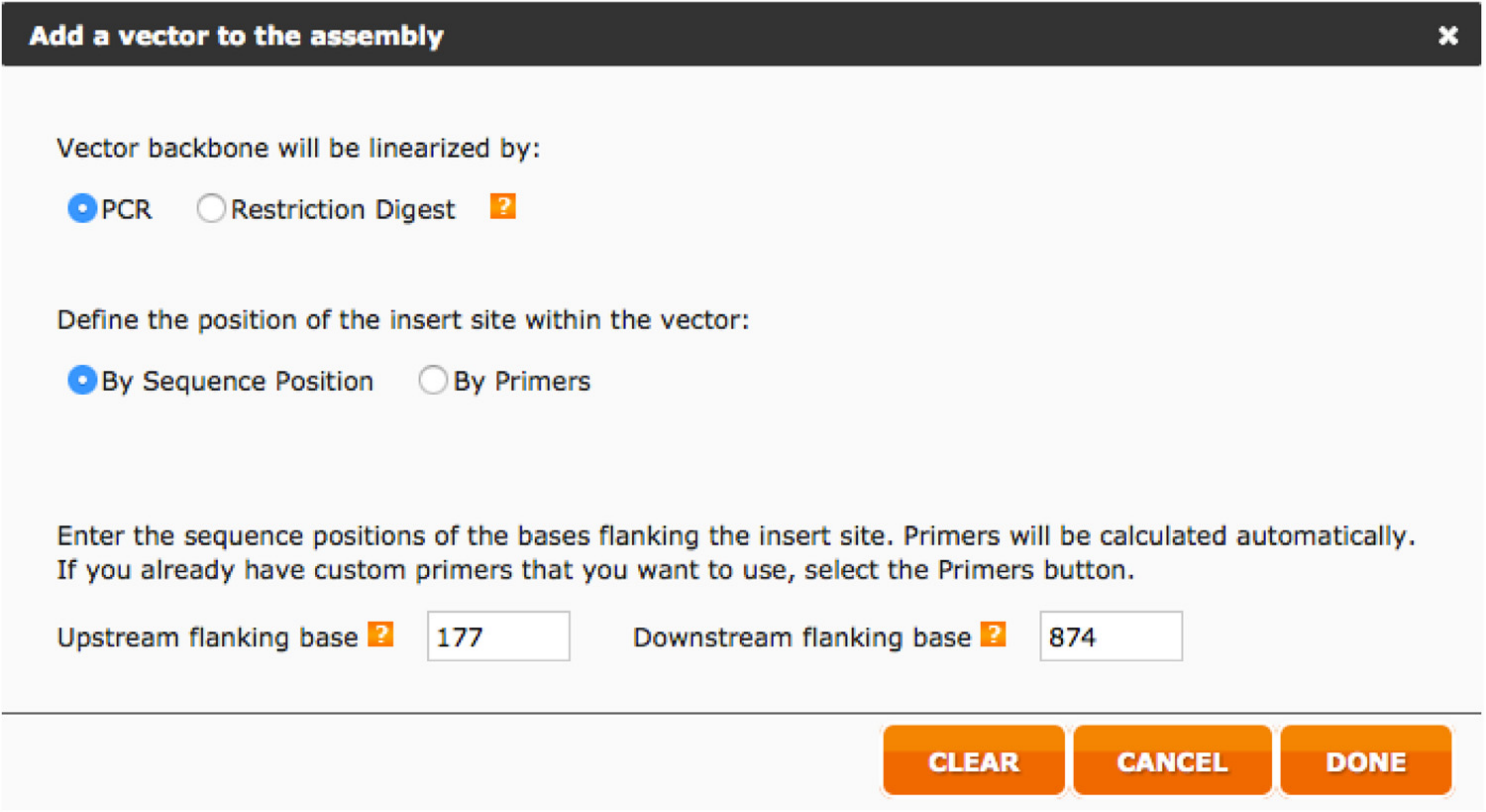
Preferences to be set for parental 2 HA (stalk donor) sequence.

**Fig. 5:**
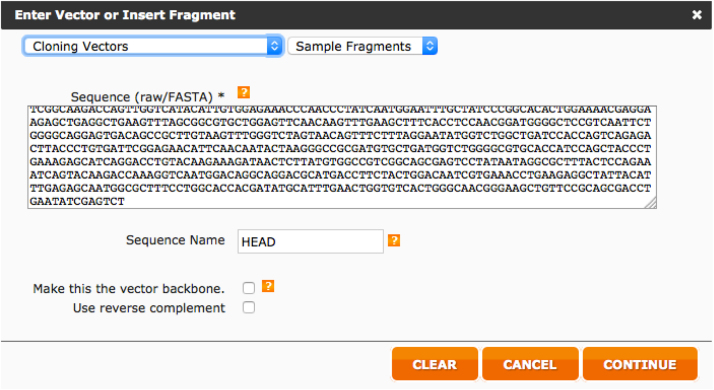
Entry of the parental 1 HA (head donor) sequence.

**Fig. 6:**
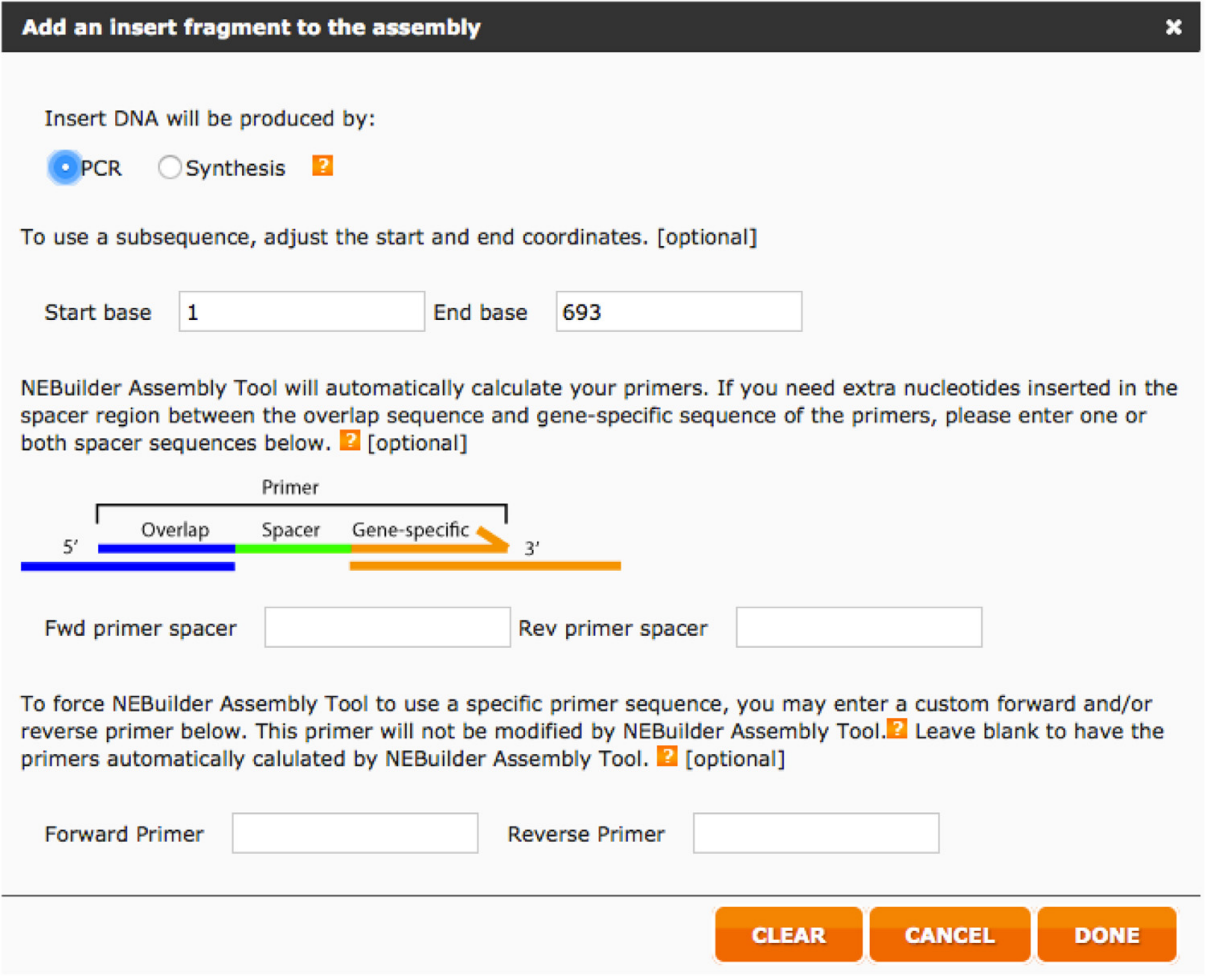
Preferences to be set for parental 1 HA (head donor) sequence.

**Fig. 7:**
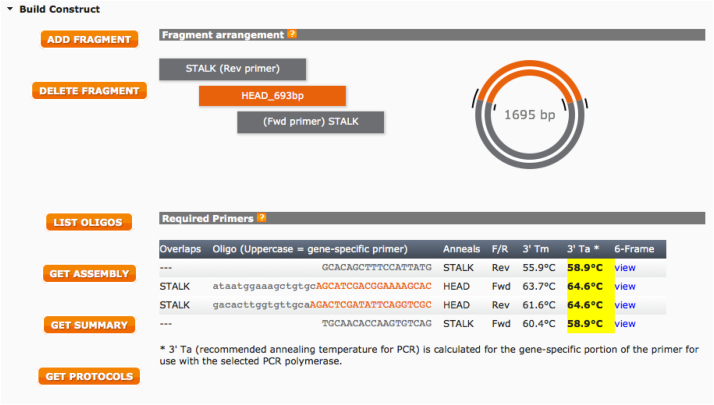
Example of designed primers. Oligonucleotide sequences to be ordered are reported in the table with information about the regions that they anneal to, and the Annealing Temperature (3′Ta) that should be used in the PCR to amplify the stalk plus vector and the head fragments.

• Open the NEBuilder Assembly Tool (http://nebuilder.neb.com/)
• In “set preferences” select E5510 Gibson Assembly Cloning kit and check that the other preferences are set as shown in Fig. 2.
• Select “**Build Construct**” and press “ADD FRAGMENT”.
• Copy in the box the complete nucleotide sequence of the *parental 2* HA, which is the one from which the stalk region is taken, name it (STALK), and select “Make this the vector backbone” as shown in Fig. 3. Press “CONTINUE”.
• In “Vector backbone will be linearized” sub-section select “PCR” as shown in Fig. 4.
• In ‘Define the position of the insert site within the vector’ sub-section select “By Sequence Position” as shown in Fig. 4.
• Now insert in the “Upstream flanking base” and “downstream flanking base” sub-sections the numbers previously identified in step V above. Then press “DONE”.
• Press “ADD FRAGMENT” and copy the nucleotide sequence of the *parental* 1 HA head. Remember to name the sequence (HEAD) as shown in Fig. 5, and then press “CONTINUE”.
• In “Insert DNA will be produced by” sub-section select “PCR” as shown in Fig. 6. Leave all the other values untouched and then press “DONE”.
• The tool will automatically design the primers and additionally advise on the annealing temperature to use as shown in Fig. 7. The software also provides the assembled sequence, that once translated should be checked by reference to the predicted amino acid sequence of the cHA designed in step IV.
• Primers can be ordered from a range of commercial suppliers desalted and at 25 nm scale or lower.

### Step 2: PCR

PCR of the head fragment and of the stalk plus vector fragment should be performed following the manufacture’s instruction using Q5^®^ High-Fidelity DNA Polymerase. We routinely perform minor changes, such as increasing the denaturation/annealing and extension times. In Table 1, Table 2, the thermocycler protocols used for amplification of the HA stalk plus vector and the HA head fragments are reported.

### Step 3: Analytical gel electrophoresis

**Fig. 8:**
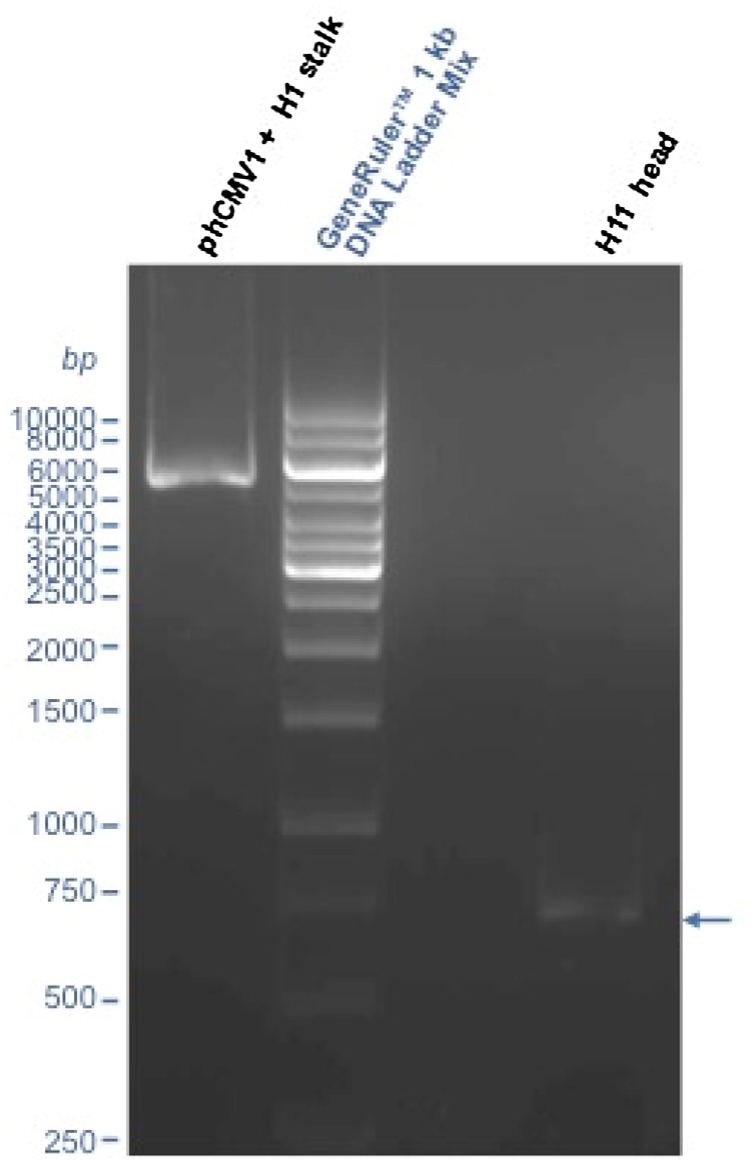
Amplification of HA stalk plus vector and HA head. In the agarose gel, two bands corresponding to the amplified product of the HA stalk plus vector of phCMV1-A/South Carolina/1/1918 H1 and of the HA head of phCMV1-A/duck/Memphis/546/1974 H11 can be observed.

5 μl of PCR reaction should be run on a 0.7–1% agarose gel to confirm the amplification of the stalk plus vector and of the head region (Fig. 8). To correctly perform agarose gel electrophoresis we recommend consulting “Molecular Cloning: A Laboratory Manual” [7].

**Note:** if preferred the two PCR reactions can be run on a preparative gel to then proceed to gel extraction of the DNA bands corresponding to fragments amplified. In this case STEP 4–6 are not required. We do not use gel extraction, as in our hands using commercial kits, the efficiency of gel extraction is lower than PCR purification.

### Step 4: PCR purification

When the amplification of the target DNA has been confirmed, the PCR reaction must be purified before proceeding to additional steps (e.g. *DpnI* digestion or Gibson Assembly). QIAquick PCR Purification Kit (QIAGEN) or similar can be used for this purpose. DNA concentration should be measured using Nanodrop instruments or a spectrophotometer.

### Parental DNA digestion using *DpnI* (recommended)

To increase Gibson Assembly efficiency and reduce background from residual template DNA, restriction enzyme digestion using *DpnI* can be performed. *DpnI* is a restriction enzyme that can cleave DNA at a specific site (GATC), but only if this is methylated or hemimethylated. This permits the degradation of plasmid template DNA isolated from *dam+ E. coli* strains but not of the amplified PCR products. FastDigest^®^ *DpnI* can be used following the manufacturer’s instructions. Routinely 400–600 ng of purified PCR reaction is digested in a total volume of 10–20 μl (depending on the DNA concentration) using 1 μl of enzyme (10U). The digestion reaction is performed for 15 min at 37 °C in a water bath or ideally in a heating block pre-set to 37 °C. Subsequently the reactions should be purified to remove buffer salts and enzymes and for subsequent use in the Gibson assembly reaction.

### Step 6: Gibson assembly

Gibson assembly must be performed following manufacturer’s instructions: the reaction is performed in a 20 μl total volume in which 10 μl Gibson Assembly Master Mix, vector and insert, and DNase and RNAse are added. Good results have been obtained using a vector (HA stalk plus vector):insert (HA head) ratio 1:3. For example, for a HA stalk plus vector of ∼5.3 Kb and a head fragment of ∼700 bp, 80 ng of HA stalk plus vector, and 29 ng HA head should be used in the reaction. The Gibson assembly reaction should be incubated for 15 min at 50 °C, before proceeding to bacterial transformation following manufacturer’s instructions.

### Step 7: Colony PCR screening (optional)

If numerous colonies are present on the plate of the transformed Gibson assembly reaction, it is possible to perform colony PCRs to simultaneously screen more clones for the presence of the assembled corrected HA. For this purpose vector specific primers or gene specific primers can be used. Vector specific primers should anneal upstream and downstream of the cHA gene and they can potentially amplify the parental HA (especially the parental stalk HA since its encoding plasmid is always the same as the cHA) if the *DpnI* digestion was not performed correctly. Gene specific primers can be designed as an alternative: one should anneal the cHA stalk, and the other the cHA head. Primers used for amplification of the HA head can also be used, but will result in less informative screening (since it is possible to amplify the parental HA, if its encoding plasmid was not correctly digested using *DpnI*). It is extremely important that during colony PCR all the tubes are always numbered correctly to be able to identify positive clones.

Colony PCR can be performed as follows.

i A PCR stock mix can be prepared by considering the number of samples to be screened and for each reaction adding 12.5 μl of DreamTaq Green PCR Master Mix, 0.1 μl of each Fwd and Rev primers (final concentration 400 nM).
ii Subsequently the PCR stock mix should be aliquoted into PCR microtubes (20 μl in each tube).
iii Alongside the PCR stock mix preparation, each bacterial colony to be tested should be numbered and diluted in 20 μl of DNase/RNase free water before streaking the colony onto a numbered grid previously prepared on an appropriate LB Agar plate.
iv Negative (colony with empty vector) and positive (colony with vector and insert) controls are inserted if available.
v An additional control consisting of DNase/RNase free water can be added to evaluate if carry‐over of DNA was present during the procedure.
vi After a lysis step at 94 °C for 3 min in a thermal cycler, 5 μl of each colony suspension is transferred to a numbered PCR microtube in which 20 μl of PCR mix is already present.
vii Tubes should be positioned in a thermocycler and the colony PCR program detailed in Table 3 should be run.
viii Amplification of the target sequence should be verified through analytical DNA gel electrophoresis. If DreamTaq Green PCR Master Mix was used samples can be directly loaded on an agarose gel.

If vector specific primers were used the positive clones to be further analysed are the ones in which a 1.7 Kbp band is present after analytical DNA gel electrophoresis. For gene specific primers the length of the band will be dependent on the position of the primer used. If primers annealing to stalk and head of the cHA were used, amplification should be observed only for the clones in which the cHA was correctly assembled. Positive colonies from the plate streaked during the colony PCR can be selected for further analysis in step 8.

### Step 8: Isolation of plasmid dna

(Positive) colonies resulting from bacterial transformation of the Gibson assembly reaction should be inoculated and plasmid DNA should be isolated using QIAprep Spin Miniprep Kit following manufacturer’s instructions.

### Step 9: Screening with restriction enzymes and Sanger sequencing

This step is technically demanding: it is necessary to identify single, or a couple of restriction enzymes that cleave with differential patterns the two parental HAs and the newly generated cHA plasmids. Where possible full plasmid sequence should be analysed but, if not available, the analysis can be performed using HA sequences, the empty plasmid sequence, and the enzyme sites used for the original cloning. To identify the enzymes, software such as DNADynamo that permits virtual digestion of sequences can be used to test different enzyme combinations and calculate the size of the DNA bands expected after electrophoresis. For each possible enzyme or couple of enzymes the band patterns observed after electrophoresis should be analysed for the two parental HAs and the newly generated cHA plasmids. Once the appropriate restriction enzymes are identified, plasmid clones should be digested. Plasmid DNA digestions of the Gibson assembly reaction clones and of the two parental HAs should be performed following the manufacturer’s instructions of the restriction enzyme used. The use of FastDigest™ enzymes using the FastDigest™ Green Buffer for direct loading on gels is recommended. Usually 600 ng of DNA and a 15 min incubation at 37 °C can be used for optimal results. Reactions directly loaded on a 1% agarose gel can be subjected to gel electrophoresis using TAE buffer. Undigested plasmid, especially of the parental HA, should also be added as control in the gel electrophoresis. After running the gel at an appropriate voltage and for an appropriate amount of time, the gel should be visualized using a gel doc system and the bands should be identified. Clones that show a digestion pattern ascribable to cHA should be subjected to Sanger sequencing using primers that anneal to the vector upstream and downstream of the cHA gene for final confirmation.

### Step 10: Sequence identification

Amino acid identity of the cHA construct with the theoretical amino acid sequence generated using the NEBuilder Assembly Tool should be verified after Sanger sequencing of the selected clones before using the constructs for further applications. The assembled sequence is generally correct, however single point mutations (probably introduced during PCR) can be observed in certain clones.

## Pseudotype particle production

Lentiviral or retroviral pseudotypes harbouring the cHA with different vector profiles can be produced depending on the core proteins and genome expressing plasmid selected. Some further points that should be considered before performing production or optimization of pseudotypes are presented below.

○ If the stalk of a highly pathogenic avian influenza haemagglutinin (H5 or H7), which possess a multibasic cleavage site, was used to generate the cHA it is possible to produce pseudotype particles as previously described [8], [9] using a transfection DNA mix containing a lentiviral (or retroviral) vector and a packaging system (or pNL4.3), and the cHA encoding plasmid.
○ Alternatively, if the stalk originates from a human (seasonal) or a low pathogenic avian influenza haemagglutinin, possessing a single arginine at the cleavage site, a protease-encoding plasmid should be added to the transfection mix. As previously reported, it is necessary to optimize quantity and type of proteases used.

**Fig. 9:**
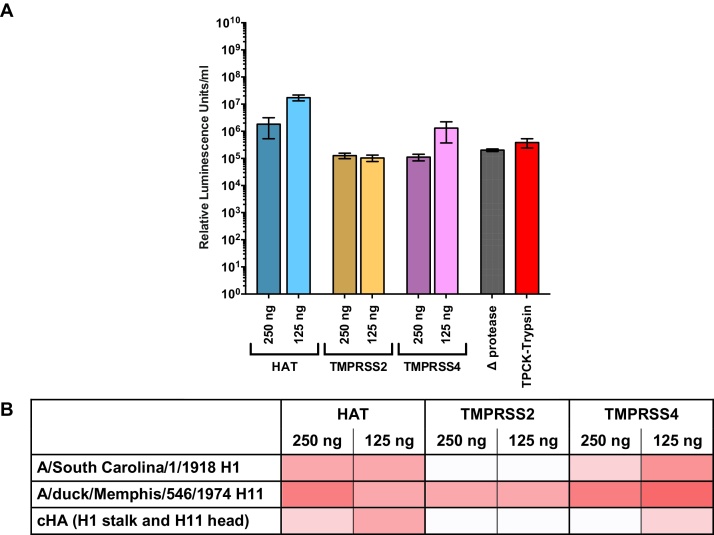
Production of chimeric haemagglutinin pseudotypes.

**Note:** It has been observed that the cleavage conditions used to produce pseudotype particles harbouring cHAs do not correspond to the cleavage conditions of the parental HA that has donated the stalk and it is not yet possible to predict the conditions that need to be used (Fig. 9).

• Additional and concurrent optimizations of the quantity of the HA plasmid transfected for production of pseudotypes can also be necessary in certain cases.
• Exogenous neuraminidase (Sigma-Aldrich or Roche) should be added to facilitate pseudotype release 24 h post-transfection. Alternatively, a neuraminidase encoding plasmid can be added to the transfection mix.

A protease encoding plasmid is added to the transfection mix to enable HA activation when the parental HA used for the stalk posseses a monobasic cleavage site. Different types of proteases and different quantities of plasmids need to be tested to obtain higher titre pseudotype particles. Pseudotypes produced in absence of a protease-encoding plasmid should be used as controls. These pseudotypes can also be activated using TPCK-trypsin. **A.** As an example the production titres obtained for a cHA with H1 stalk and H11 head are reported. **B.** Comparison between the activiation profiles of pseudotype with cHA (H1 stalk and H11 head) or the parental HAs: the darker color indicates higher titer.

## Pseudotype neutralization assay

A cHA pseudotype neutralization assay should be performed as previously reported [11]. Data can be analysed to calculate the half maximal inhibitory concentration (IC_50_) using statistical software such as Graphpad Prism or Microsoft Excel. Since the presence of homosubtypic and heterosubtypic antibodies against head epitopes cannot be excluded (since monoclonal antibodies with this activity have been isolated) additional controls should be introduced: assays using pseudotypes harbouring each of the parental HAs must be performed in parallel. This permits a holistic and comprehensive interpretation of the data and of the IC_50_ results.

Results can be interpreted following the following rationale:

• Stalk-directed responses are present if the antibody titre obtained against the cHA is greater than the titre against the parental head HA. In fact, increases in antibody titres are detected since with the cHA a conserved stalk epitope is introduced.
• If the titres against the cHA are lower than the ones against the stalk parental HA, cross-reactive antibody directed against the HA head are present. In other words, the presence of the stalk epitope does not explain the antibody titres observed using the stalk parental HA.

## Additional information

### Protein folding

It has been observed that it is difficult to evaluate and predict if the cHA created will have correct folding or will be efficiently expressed, especially if highly divergent stalk and head regions are used. For this reason, if viable pseudotypes cannot be produced, the expression of the cHA should be evaluated using immunofluorescence and/or SDS-PAGE and Western Blotting.

### Epitopes at the hinge of stalk and head regions

While enabling the identification of antibodies against the HA stalk, cHAs can prevent the detection of antibodies directed against epitopes situated between the head and the stalk regions. These epitopes may have some potential for cross-neutralization. This should be taken into consideration when data and IC_50_ results are interpreted. Furthermore, it cannot be excluded that this newly constituted hinge region generates a new epitope, which may or may not be recognised in neutralization assays.

### Flexibility

The method described permits the simple cloning of cHAs possessing the same stalk but different heads. In fact, during primer design overlapping sequences are inserted on the head fragments. For this reason, the same HA stalk plus vector can be used for multiple reactions. This could be an important advantage considering that the current pre-clinical vaccination strategy using cHAs consists of the immunisation of different cHAs that possess different heads but have common stalks [10]. The H11/H1 chimeric HA pseudotype described in Fig. 9 has been used recently by us to measure the neutralizing antibody response directed against the H1 stalk elicited by a novel dissolvable microneedle vaccine [11]. Additionally, the Gibson assembly cloning procedure that we have reported could be used with appropriate modification by other investigators to evaluate chimeric proteins. The method is extremely useful when small or medium portions of a protein, such as single epitopes or domains, need to be modified: in fact mutagenesis techniques are often not efficient and usually appropriate restriction enzyme sites to proceed with a traditional cloning step are not available. Furthermore, by adding additional sequences during the primer design and the PCR step (or through gene synthesis of a fragment in which overlapping sequence resulting from the primer design were added) it is possible to create fusion proteins when linker regions with appropriate restriction enzyme sites are not available.

## Figures and Tables

**Table 1:** PCR program for the amplification of HA stalk plus vector.

Cycles	Temperature	Time	Step
	98 °C	3 min	Initial denaturation
30 cycles	98 °C	30 s	Denaturation
	Recommended Primer Annealing Temperature	30 s	Annealing
	72 °C	6 min	Extension
	72 °C	8 min	Final extension
	4 °C	To preserve reactions until removed	

**Table 2:** PCR program for the amplification of the HA head.

Cycles	Temperature	Time	Step
	98 °C	3 min	Initial denaturation
30 cycles	98 °C	15 s	Denaturation
	Recommended Primer Annealing Temperature	30 s	Annealing
	72 °C	1 min	Extension
	72 °C	2 min	Final extension
	4 °C	To preserve reactions until removed	

**Table 3:** Colony PCR program.

Cycles	Temperature	Time	Step
	94 °C	2 min	Initial denaturation
30 cycles	94 °C	30 s	Denaturation
	Depending on Primers’ Annealing Temperature	1 min	Annealing
	72 °C	2 min	Extension
	72 °C	5 min	Final extension
	4 °C	To preserve reactions until removed	
